# Supplementary material for: Family-based exome sequencing combined with linkage analyses identifies rare susceptibility variants of MUC4 for gastric cancer
Source: PLoS One. 2020 Jul 23;15(7):e0236197. doi: 10.1371/journal.pone.0236197 (PMC7377420; doi:10.1371/journal.pone.0236197)
Supplement: S1 Fig — (PDF) [file pone.0236197.s001.pdf]

Supplementary Figure S1 Motif search was performed using MotifFinder tool

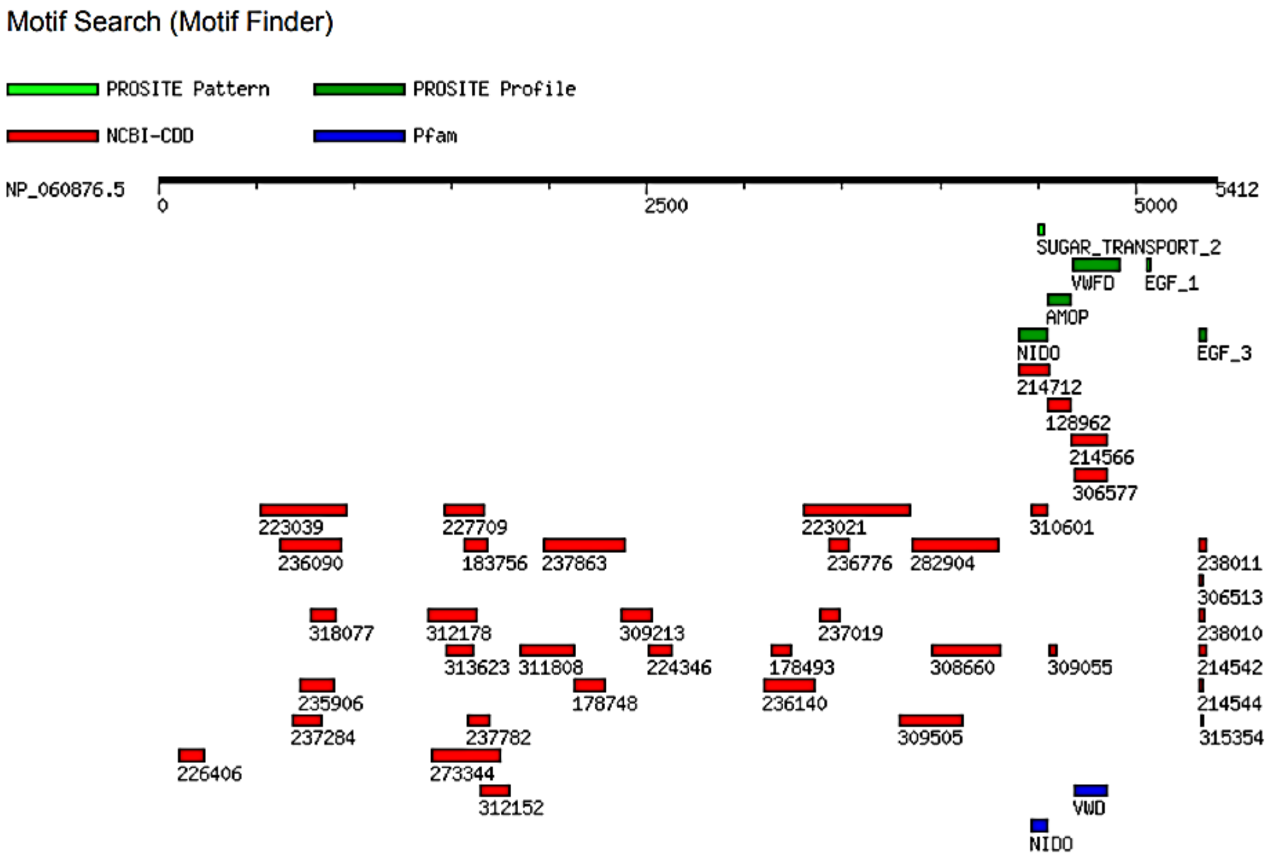

Location	Amino acid	Motif
**chr3:195512387**	**L2022F**	Motif ID 11808 loci1856..2125(38.3, 0.11)<br>pfam08017, Fibrinogen_BP, Fibrinogen binding protein.<br>Proteins in this family bind to fibrinogen. Members of this family includes the fibrinogen receptor, FbsA, which mediates platelet aggregation.
chr3:195510793	A2553V	Motif ID 224346 Loci 2509..2627(38.2, 0.19)<br>COG1429, CobN, Cobalamin biosynthesis protein CobN and related Mg-chelatases [Coenzyme metabolism].
**chr3:195475923**	**T5295M**	NA
chr3:195507271	T3727S	MotifID 223021 Loci 3301..3850(61.5, 2e-08)<br>PHA03247, PHA03247, large tegument protein UL36; Provisional.
chr3:195507778	A3558V	Part of same motif as chr3:195507271
**chr3:195513076**	**R1792H**	Motif ID 312152 loci 1649..1797(35.8, 0.93)<br>pfam08549, SWI-SNF_Ssr4, Fungal domain of unknown function (DUF1750).<br>This is a fungal domain of unknown function.<br>* insignificant finding
chr3:195513446	S1669G	Motif ID 273344 Loci 1396..1753(36.1, 0.70)<br>TIGR00927, retinal_rod, K+-dependent Na+/Ca+ exchanger.
chr3:195510803	P2550T	Part of same motif as chr3:195510793
chr3:195511811	P2214T	Motif ID 237863 Loci 1971..2385(41.2, 0.020)<br>PRK14949, PRK14949, DNA polymerase III subunits gamma and tau; Provisional.
chr3:195511813	S2213N	Part of same motif as chr3: 195511811
