# Supplementary figures and images for: Family-based exome sequencing combined with linkage analyses identifies rare susceptibility variants of MUC4 for gastric cancer
Source: PLoS One. 2020 Jul 23;15(7):e0236197. doi: 10.1371/journal.pone.0236197 (PMC7377420; doi:10.1371/journal.pone.0236197)

## Supplementary Figure S2 QQ plots for LOD p-values of three different gene size groups

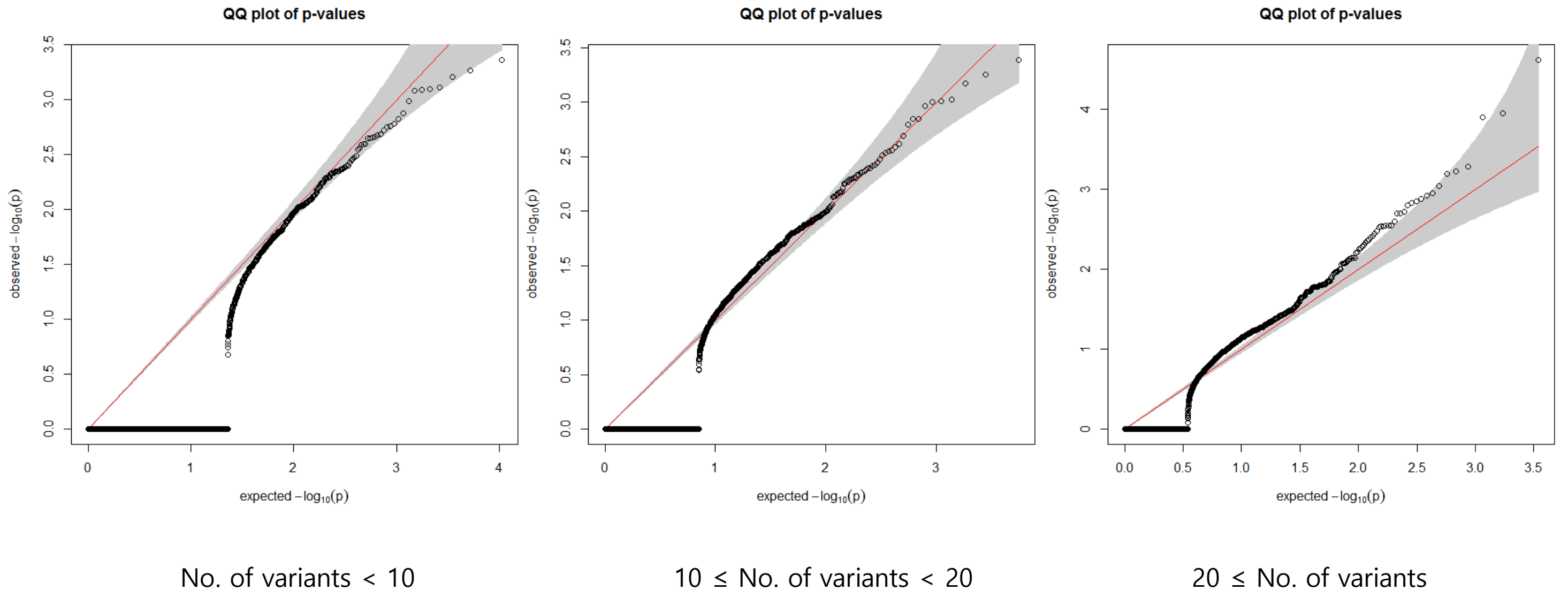

Supplement: S2 Fig — (PDF) [file pone.0236197.s002.pdf]
