# Supplementary material for: Family-based exome sequencing combined with linkage analyses identifies rare susceptibility variants of MUC4 for gastric cancer
Source: PLoS One. 2020 Jul 23;15(7):e0236197. doi: 10.1371/journal.pone.0236197 (PMC7377420; doi:10.1371/journal.pone.0236197)
Supplement: S3 Fig — (PDF) [file pone.0236197.s003.pdf]

Supplementary Figure S3: GWAS analysis

Gastric cancer patients (n = 597), healthy individuals (n = 9759)

1) *MUC4* region (chr3:195,473,637-195,539,149) including 0.5MB flanking region

CHR	SNP	BP	P	OR	L95	U95	A1	A2	MAF_A	MAF_U	MAF_DB	HWE	MISS
3	rs148735556	195052426	1.27e-07	4.343	2.519	7.487	T	A	0.0235	0.0054	0.0113	0.3515	0
3	rs11717039	195401737	1.071e-05	1.386	1.198	1.603	C	T	0.4782	0.4026	0.4534	0.07002	0

<SNP Table> SNP lists whose p-value is significant

*0.05 / 4224 = 1.18e^{-5}

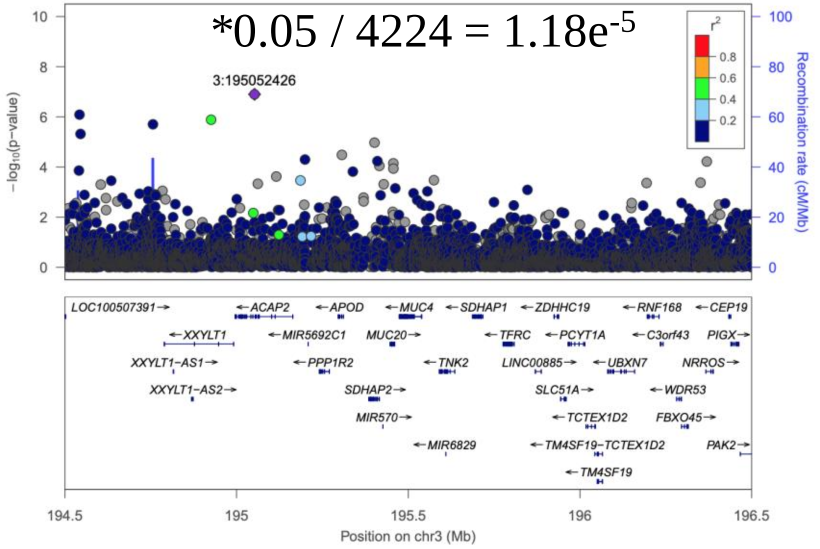

2) *MUC4* region (exon 2 and exon 24)

Exon	SNP	BP	AA change	Annotation	P	OR	L95	U95	A1	A2	MAF_A	MAF_U	HWE	MISS
2	rs547775645	195515398	p.Ser1018Cys	missense	1.12e-03*	17.75	3.148	100.1	C	G	0.0050	0.0004	1	0

<SNP Table> SNP lists whose p-value is significant

*0.05 / 25 = 2e^{-3}
