# Supplementary material for: Family-based exome sequencing combined with linkage analyses identifies rare susceptibility variants of MUC4 for gastric cancer
Source: PLoS One. 2020 Jul 23;15(7):e0236197. doi: 10.1371/journal.pone.0236197 (PMC7377420; doi:10.1371/journal.pone.0236197)

Supplementary Figure 4

A predicted structure of T5295M (chr3:195475923) using Structure Prediction Tool Phyre2. T5295M encoding region was a loop region between the 2nd and 3rd EGF domain of MUC4α. Especially, the third EGF-like domain of MUC4 was modeled by Modeller 9v10. Chain D from LRP4 complex structure (PDB Id: 3v65) was used as a template for homology modelling which was searched by Swissmodel server. The SNP loci is also near or part of N-glycosylation cite with the N-glycosylation pattern of N-Xaa-ST→ML.

The red sphere denotes SNP loci and predicted EGF domains are color coded in blue, sky blue, and orange.

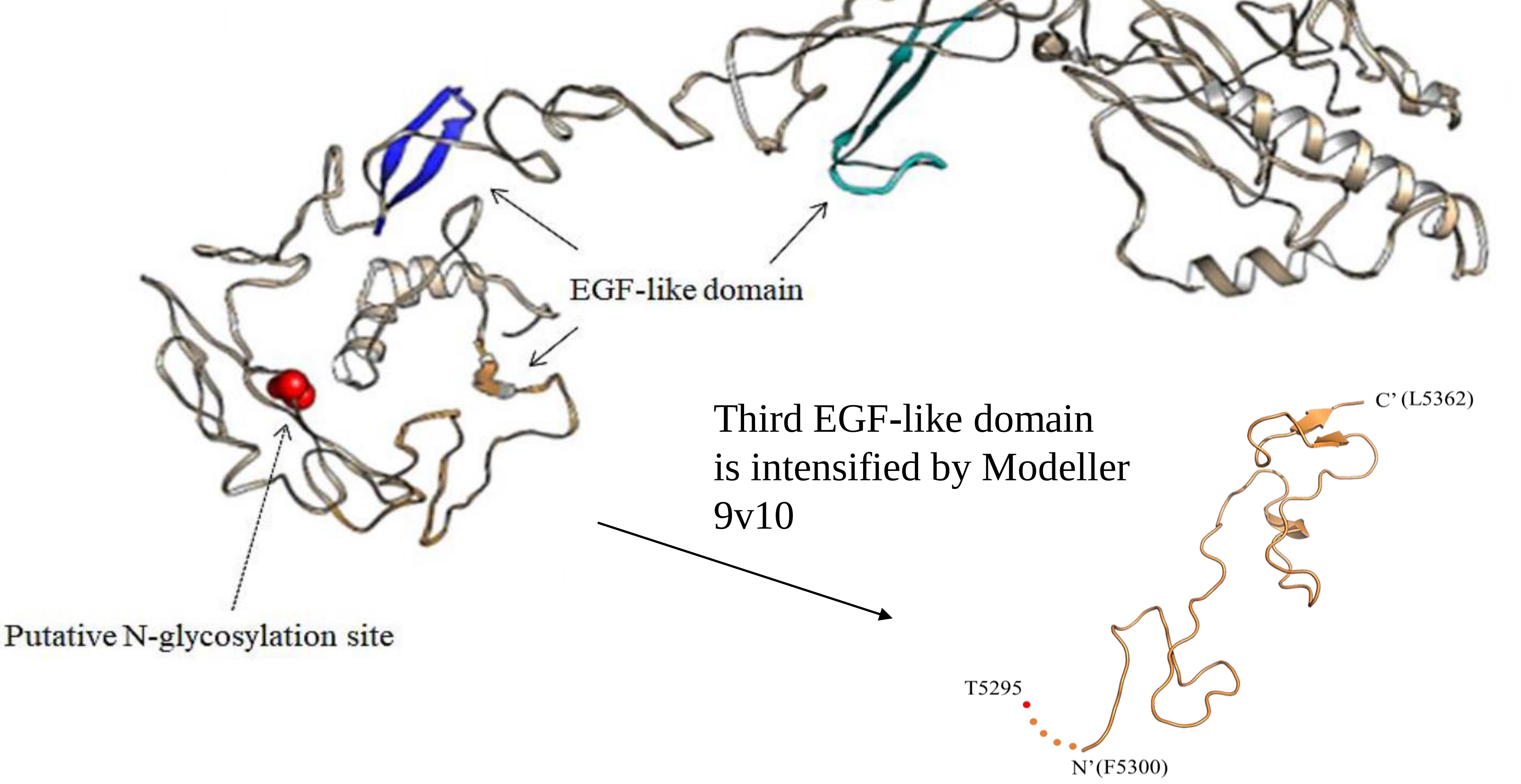

Supplement: S4 Fig — T5295M encoding region was a loop region between the 2nd and 3rd EGF domain of MUC4 α. Especially, the third EGF like domain of MUC4 was modeled by Modeller 9v10. Chain D from LRP4 complex structure (PDB Id: 3v65) was used as a template for homology modelling which was searched by Swissmodel server. The SNP loci is also near or part of N glycosylation cite with the N glycosylation pattern of N Xaa ST→ML. The red sphere denotes SNP loci and predicted EGF domains are color coded in blue, sky blue, and orange. (PDF) [file pone.0236197.s004.pdf]
