# Supplementary material for: Family-based exome sequencing combined with linkage analyses identifies rare susceptibility variants of MUC4 for gastric cancer
Source: PLoS One. 2020 Jul 23;15(7):e0236197. doi: 10.1371/journal.pone.0236197 (PMC7377420; doi:10.1371/journal.pone.0236197)
Supplement: S1 Table — (PDF) [file pone.0236197.s005.pdf]

Supplementary Table S1. Characteristics of 55 participants in study

Fam	ID	Sex	Age[a]	GC	Path[b]	HDGC[c]	HP	Erad[d]	*MUC*4	MUC1[e]	Smoking	Alcohol	Note
1	1	M	63	N			(+)	S	N	AA	Y	Y	
1	2	M	60	Y	I	N/A	(-)	N	Y	AA	N	Y	
1	3	M	58	N			(-)	N	N	AA	Y	Y	
1	4	M	72	N			(+)	F/N	N	AA	Y	Y	
2	5	F	53	Y	I	N	(+)	S	Y	AA	N	N	
2	6	F	60	N			(+)	F/N	N	AA	N	N	
2	7	F	65	N			(+)	F	N	AA	N	N	
2	8	F	70	N			(+)	F/N	N	AA	N	Y	
3	9	F	58	N			(+)	S	N	AA	Y	N	
3	10	F	70	N			(+)	F/N	N	AA	N	N	
3	11	F	84	Y	I	N	(-)	N	Y	AA	N	N	
4	12	F	68	N			(+)	S	N	AA	N	N	
4	13	F	79	N			(+)	S	N	AA	N	N	
4	14	M	77	N			(+)	F/N	N	GA	N	Y	
4	15	M	54	Y	N/A	N/A	(-)	N	Y	AA	Y	Y	
4	16	F	71	N			(+)	S	Y	GA	N	N	
4	17	F	80	N			(+)	S	N	AA	N	N	
5	18	M	50	N			(+)	S	N	AA	N	Y	
5	19	M	71	Y	M	N/A	(-)	N	Y	AA	Y	Y	
6	20	M	56	Y	I	N/A	(+)	S	N	AA	Y	Y	
6	21	M	69	N			(+)	S	N	AA	N	Y	
6	22	F	62	N			(-)	N	N	AA	N	N	
7	23	F	50	Y	D	Y	(+)	S	Y	AA	N	Y	*CTNNA1*[f]
7	24	M	51	N			(+)	S	N	AA	Y	Y	
7	25	F	61	N			(+)	F/N	N	AA	N	Y	
7	26	F	54	N			(+)	F/N	N	AA	N	Y	
7	27	M	58	N			(-)	N	N	GA	Y	Y	
8	28	M	39	Y	D	Y	(-)	N	N	AA	N	Y	*CDH1*[g]
8	29	F	50	N			(-)	N	N	AA	N	Y	
9	30	M	60	Y	I	N	(+)	S	Y	AA	Y	Y	
9	31	M	60	N			(-)	N	N	AA	Y	Y	
10	32	M	56	Y	I	N	(+)	S	Y	GG	Y	Y	
10	33	M	59	Y	I	N	(+)	S	Y	GG	Y	N	
11	34	F	78	Y	I	N/A	(+)	S	Y	AA	Y	Y	
11	35	F	51	N			(+)	F/N	N	AA	N	Y	
11	36	F	67	N			(+)	F/N	N	GA	N	Y	

11	37	F	65	N			(+)	F/N	Y	GA	N	N	
11	38	F	68	Y	N/A	N	(-)	N	Y	AA	N	N	
11	39	F	69	N			(+)	F/N	Y	AA	N	N	
11	40	M	54	N			(+)	S	N	AA	N	Y	
11	41	F	50	N			(-)	N	N	AA	N	Y	
11	42	F	56	N			(+)	F/N	N	AA	N	Y	
12	43	F	80	Y	I	N	(+)	F	N	AA	N	N	
12	44	F	63	N			(-)	N	N	AA	N	N	RCC
12	45	M	40	Y	I	N	(-)	N	N	AA	Y	Y	RCC
13	46	F	31	Y	D	Y	(+)	S	N	AA	Y	Y	
13	47	F	51	N			(+)	S	N	AA	Y	Y	
13	48	F	50	N			(-)	N	N	AA	N	N	
13	49	F	77	N			(+)	S	N	AA	Y	N	
14	50	F	69	N			(+)	N	N	AA	N	Y	
14	51	M	66	Y	I	N	(-)	N	Y	AA	Y	Y	
14	52	M	60	Y	I	N	(+)	S	Y	AA	Y	Y	
14	53	M	58	Y	I	N	(+)	S	Y	AA	Y	Y	
14	54	M	56	N			(-)	N	N	AA	Y	Y	
14	55	M	54	N			(+)	N	N	AA	N	Y	

[a] Age at diagnosis of gastric cancer was presented for gastric cancer patients while others are ages of at the enrollment.

[b] Based on Lauren classification

[c] International Gastric Cancer Linkage Consortium 2010 clinical criteria: Family with 2 or more cases of gastric cancer with at least 1diffuse gastric cancer diagnosed before the age of 50 years old.

[d] S, eradicated; F, anti-*H. pylori* therapy was performed but failed; N, anti-*H. pylori* therapy was not performed; F/N, either F or N (based on antibody value in the absences of treatment information)

[e] MUC1:rs4072037

[f]NM_001323994:exon5:c.A283G:p.I95V

[g]NM_001317184:exon8:c.G1057A:p.E353K

Fam., number of family; ID, identification; Path, pathology; I, intestinal-type; D, diffuse-type; M. mixed-type; GC, gastric cancer; HDGC, hereditary gastric cancer syndrome; RCC, renal cell cancer; HP, *H. pylori*; M, male; F, female; no., number; Y, yes; N/A, not available; (+), positive; (-), negative
