# Supplementary material for: Family-based exome sequencing combined with linkage analyses identifies rare susceptibility variants of MUC4 for gastric cancer
Source: PLoS One. 2020 Jul 23;15(7):e0236197. doi: 10.1371/journal.pone.0236197 (PMC7377420; doi:10.1371/journal.pone.0236197)
Supplement: S2 Table — (PDF) [file pone.0236197.s006.pdf]

Supplementary Table S2. Summary of whole exome sequencing quality control statistics

Data /Sample	Total reads	Total yield (bp)	Average read length (bp)	Target regions (bp)	Average throughput depth of target regions (X)	Initial mappable reads (mapped to human genome)	% Initial mappable reads	Non-redundant reads	% Non-redundant reads	On-target reads	% On-target reads	% Coverage of target regions (more than 1X)	% Coverage of target regions (more than 10X)	% Coverage of target regions (more than 20X)	Mean depth of target regions (X)
1	95,612,806	9,656,893,406	101	60,456,963	159.7	95,114,119	99.4	92,383,992	97.1	65,376,265	70.7	99.9	98.9	96.7	92.2
2	92,742,526	9,366,995,126	101	60,456,963	154.9	92,137,405	99.3	88,891,738	96.4	63,881,541	71.8	99.9	98.9	96.7	90
3	125,133,614	12,638,495,014	101	60,456,963	209	124,295,344	99.3	119,496,950	96.1	83,535,213	69.9	99.9	99.3	98	117.5
4	97,625,162	9,860,141,362	101	60,456,963	163	96,900,187	99.2	94,046,228	97	65,127,212	69.2	99.9	99	96.9	91.4
5	108,883,104	10,997,193,504	101	60,456,963	181.9	108,707,802	99.8	105,474,454	97	76,254,888	72.2	99.6	98.9	97.3	107.9
6	126,752,254	12,801,977,654	101	60,456,963	211.7	126,478,759	99.7	122,332,240	96.7	86,448,767	70.6	99.6	99	97.6	122.3
7	111,119,786	11,223,098,386	101	60,456,963	185.6	110,846,255	99.7	106,745,981	96.3	74,355,263	69.6	99.6	98.9	97.3	105.1
8	104,493,142	10,553,807,342	101	60,456,963	174.5	104,269,176	99.7	101,244,317	97	70,701,115	69.8	99.6	98.8	97	99.8
9	105,793,398	10,685,133,198	101	60,456,963	176.7	105,564,599	99.7	102,596,515	97.1	70,035,882	68.2	99.6	98.8	97.1	98.8
10	110,554,894	11,166,044,294	101	60,456,963	184.6	110,317,455	99.7	105,908,869	96	75,353,138	71.1	99.6	99	97.5	107
11	91,996,334	9,291,629,734	101	60,456,963	153.6	91,824,193	99.8	89,158,184	97	62,819,878	70.4	99.6	98.7	96.5	89
12	96,431,574	9,739,588,974	101	60,456,963	161	95,844,326	99.3	92,940,714	96.9	65,827,721	70.8	99.6	98.7	96.6	92.8
13	89,708,038	9,060,511,838	101	60,456,963	149.8	89,185,398	99.4	87,156,538	97.7	60,426,351	69.3	99.7	98.7	96.2	85.2
14	120,851,546	12,206,006,146	101	60,456,963	201.8	119,904,312	99.2	115,271,625	96.1	79,748,415	69.1	99.9	99.2	97.7	112
15	116,642,004	11,780,842,404	101	60,456,963	194.8	115,974,942	99.4	111,913,317	96.4	79,062,263	70.6	99.9	99.3	97.8	111.6
16	103,409,598	10,444,369,398	101	60,456,963	172.7	102,757,408	99.3	99,168,572	96.5	69,893,851	70.4	99.6	98.9	97	98.4

17	108,656,888	10,974,345,688	101	60,456,963	181.5	108,170,147	99.5	105,643,540	97.6	74,217,958	70.2	99.7	98.9	97.3	105
18	118,510,578	11,969,568,378	101	60,456,963	197.9	118,004,114	99.5	114,792,761	97.2	81,751,632	71.2	99.9	99.3	97.9	115.6
19	100,962,996	10,197,262,596	101	60,456,963	168.6	100,380,264	99.4	98,128,016	97.7	67,805,716	69	99.9	99.1	97.2	95.5
20	118,896,046	12,008,500,646	101	60,456,963	198.6	118,294,024	99.4	115,220,054	97.4	79,198,085	68.7	99.9	99.3	97.9	111.6
21	103,787,766	10,482,564,366	101	60,456,963	173.3	103,246,496	99.4	100,423,139	97.2	71,021,071	70.7	99.9	99.1	97.3	100.1
22	108,287,326	10,937,019,926	101	60,456,963	180.9	107,795,941	99.5	105,121,927	97.5	72,555,138	69	99.7	99	97.4	102.3
23	83,916,752	8,475,591,952	101	60,456,963	140.1	83,491,877	99.4	81,317,219	97.3	57,922,000	71.2	99.6	98.6	96.1	81.9
24	74,194,586	7,493,653,186	101	60,456,963	123.9	73,448,651	98.9	71,112,449	96.8	51,046,038	71.7	99.9	98.3	94.1	71.6
25	107,667,122	10,874,379,322	101	60,456,963	179.8	106,499,610	98.9	103,148,605	96.8	71,488,838	69.3	99.6	98.8	97	100.5
26	91,102,234	9,201,325,634	101	60,456,963	152.1	90,774,879	99.6	88,439,685	97.4	61,412,779	69.4	99.9	98.8	96.4	86.3
27	88,693,254	8,958,018,654	101	60,456,963	148.1	88,352,947	99.6	86,030,186	97.3	58,808,150	68.3	99.9	98.8	96.1	82.7
28	88,567,950	8,945,362,950	101	60,456,963	147.9	88,258,208	99.6	86,079,155	97.5	60,647,114	70.4	99.9	98.8	96.2	85.5
29	83,749,170	8,458,666,170	101	60,456,963	139.9	83,469,716	99.6	81,709,633	97.8	56,870,551	69.6	99.6	98.3	95.5	80
30	84,691,094	8,553,800,494	101	60,456,963	141.4	84,291,125	99.5	82,211,637	97.5	56,224,325	68.3	99.9	98.7	95.8	78.9
31	80,823,074	8,163,130,474	101	60,456,963	135	80,521,204	99.6	78,702,118	97.7	54,663,477	69.4	99.9	98.5	95.3	76.8
32	84,762,346	8,560,996,946	101	60,456,963	141.6	84,402,044	99.5	82,388,206	97.6	57,114,027	69.3	99.9	98.6	95.7	80.1
33	95,899,122	9,685,811,322	101	60,456,963	160.2	95,530,781	99.6	93,155,625	97.5	66,141,420	71	99.8	98.6	96.2	93.1
34	76059798	7682039598	101	60,456,963	127	75957391	99.8	73351413	96.5	51854207	70.6	99.6	98.4	94.8	73.9
35	75950800	7671030800	101	60,456,963	126.8	75865206	99.8	73675908	97.1	52960514	71.8	99.6	98.4	95	75.5
36	75964280	7672392280	101	60,456,963	126.9	75874900	99.8	73128718	96.3	51897482	70.9	99.6	98.3	94.9	74
37	78438370	7922275370	101	60,456,963	131	78350696	99.8	75580231	96.4	53900664	71.3	99.6	98.4	95.3	76.7
38	75483912	7623875112	101	60,456,963	126.1	75384852	99.8	73269816	97.1	51048170	69.6	99.6	98.4	95	72.6

39	76450350	7721485350	101	60,456,963	127.7	76370662	99.8	73673177	96.4	53177516	72.1	99.6	98.4	94.8	75.9
40	77887486	7866636086	101	60,456,963	130.1	77779388	99.8	75106493	96.5	53325462	70.9	99.8	98.6	95.1	75.9
41	72676584	7340334984	101	60,456,963	121.4	72592105	99.8	70386625	96.9	50514832	71.7	99.6	98.2	94.1	72
42	72683254	7341008654	101	60,456,963	121.4	72615384	99.9	70196071	96.6	51657705	73.5	99.6	98.2	94.4	73.7
43	74294632	7503757832	101	60,456,963	124.1	74194318	99.8	71682333	96.6	51802122	72.2	99.6	98.4	94.9	73.9
44	71510988	7222609788	101	60,456,963	119.4	71417726	99.8	68926916	96.5	48797437	70.7	99.6	98.1	94	69.5
45	91660038	9257663838	101	60,456,963	153.1	91478919	99.8	88096926	96.3	68485960	77.7	99.8	97.5	93	98.7
46	78088726	7886961326	101	60,456,963	130.4	78006069	99.8	75279046	96.5	54415128	72.2	99.6	98.3	95.1	77.5
47	78215716	7899787316	101	60,456,963	130.6	78111854	99.8	75426375	96.5	54872893	72.7	99.6	98.4	95.1	78.2
48	77415090	7818924090	101	60,456,963	129.3	77316614	99.8	74562809	96.4	53403478	71.6	99.6	98.3	94.8	76.2
49	77705828	7848288628	101	60,456,963	129.8	77623400	99.8	75141380	96.8	55119982	73.3	99.6	98.4	95.3	78.7
50	84,254,678	8,509,722,478	101	60,456,963	140.7	84,094,549	99.8	81,269,783	96.6	60,224,093	74.1	99.6	98.3	95.2	86.5
51	85,347,392	8,620,086,592	101	60,456,963	142.5	85,213,773	99.8	82,417,482	96.7	61,847,614	75	99.9	98.7	95.8	88.8
52	82,256,588	8,307,915,388	101	60,456,963	137.4	82,099,632	99.8	78,982,167	96.2	58,550,051	74.1	99.9	98.7	95.6	83.7
53	80,423,976	8,122,821,576	101	60,456,963	134.3	80,291,187	99.8	77,859,944	96.9	58,011,007	74.5	99.9	98.5	95.2	83.3
54	84,286,848	8,512,971,648	101	60,456,963	140.8	84,142,214	99.8	81,310,808	96.6	61,908,219	76.1	99.9	98.6	95.5	89
55	85,702,324	8,655,934,724	101	60,456,963	143.1	85,530,847	99.7	82,546,537	96.5	61,943,783	75	99.9	98.6	95.4	89.1

SureSelect V6-Post was used as a Capkit.
