# Supplementary material for: Family-based exome sequencing combined with linkage analyses identifies rare susceptibility variants of MUC4 for gastric cancer
Source: PLoS One. 2020 Jul 23;15(7):e0236197. doi: 10.1371/journal.pone.0236197 (PMC7377420; doi:10.1371/journal.pone.0236197)
Supplement: S3 Table — (PDF) [file pone.0236197.s007.pdf]

Supplementary Table S3**.** Odds ratios of independent risk factors for gastric cancer by binary logistic regression

Variables [reference]	log (OR)	OR	SE	95% *CI* of OR	*Z*-value	*p*-value
Gender [female]	1.27	3.56	1.05	(0.46 to27.63)	1.21	.225
Carrying any of *MUC4* variants [no]	4.1	58.08	1.06	(7.33 to 459.97)	3.85	< **.001**
HDGC [no]	1.02	2.78	1.04	(0.36 to 21.36)	0.98	.326
Smoking (current/ex) [never]	1.17	3.22	1.00	(0.45 to 22.80)	1.17	.241

OR, odds ratio; SE, standard error; CI, confidential interval; HDGC, hereditary diffuse gastric cancer syndrome

Adjusted for sex, carrying any of *MUC4* variants, smoking, and history of hereditary diffuse gastric cancer family.

Pseudo R^2 = 0.616

Bold font indicates statistical significance.
