# Supplementary material for: Family-based exome sequencing combined with linkage analyses identifies rare susceptibility variants of MUC4 for gastric cancer
Source: PLoS One. 2020 Jul 23;15(7):e0236197. doi: 10.1371/journal.pone.0236197 (PMC7377420; doi:10.1371/journal.pone.0236197)
Supplement: S4 Table — (PDF) [file pone.0236197.s008.pdf]

Supplementary Table S4. Frequencies of 3 SNPs in multiple tumor types from TCGA

	STAD	CRC	UCEC	LUAD	LUSC
rs774527434	0.17%	0%	0%	0%	0%
rs534779185	0%	4%	0.56%	0%	0%
rs77250903	0%	0.13%	0%	0%	0%

STAD, Stomach Adenocarcinoma; CRC, colorectal cancer; UCEC, Uterine Corpus Endometrial Cancer ; LUAD, Lung Adenocarcinoma; LUSC, Lung Squamous Cell Cancer
