# Supplementary material for: Family-based exome sequencing combined with linkage analyses identifies rare susceptibility variants of MUC4 for gastric cancer
Source: PLoS One. 2020 Jul 23;15(7):e0236197. doi: 10.1371/journal.pone.0236197 (PMC7377420; doi:10.1371/journal.pone.0236197)
Supplement: S5 Table — (PDF) [file pone.0236197.s009.pdf]

Supplementary Table S5. Glycosylation site prediction using NetOGlyc and NetNGlyc

Location[a]	Amino acid change	Site prediction
chr3:195512387	p.L2022F	Positions (2020, 2021, [ ], 2023, 2024) were expected to be an O-glycosylation site.
chr3:195510793	p.A2553V	Positions (2551, 2553, 2554, 2555) were expected to be an O-glycosylation site.
chr3:195475923	p.T5295M	Near or part of the last N-glycosylation cite (N-Xaa-ST->ML) between the 2nd and 3rd EGF domain of MUC4 β -subunit out of approximately 20 N-glycosylation predicted sites.
chr3:195507271	p.T3727S	Position (3727) was expected to be an O- glycosylation site.
chr3:195507778	p.A3558V	Positions (3556, [ ] 3559, 3560) were expected to be an O-glycosylation site.
chr3:195513076	p.R1792H	Positions (1790, 1791 [ ]) were expected to be an O-glycosylation site.
chr3:195513446	p.S1669G	Positions (1668, 1669, 1670-2) were expected to be an O-glycosylation site.
chr3:195510803	p.P2550T	Positions (2548, 2549, [ ], 2551, 2552) were expected to be an O-glycosylation site.
chr3:195511811	p.P2214T	Positions (2212, 2213,[ ],2215,2216) were expected to be an O-glycosylation site.
chr3:195511813	p.S2213N	Positions (2212, 2213, 2215, 2216) were expected to be an O-glycosylation site.

[a] Chromosome position in reference genome, GRCh37/hg19

[ ] denotes possible O-glycosylation site
