# Supplementary material for: Family-based exome sequencing combined with linkage analyses identifies rare susceptibility variants of MUC4 for gastric cancer
Source: PLoS One. 2020 Jul 23;15(7):e0236197. doi: 10.1371/journal.pone.0236197 (PMC7377420; doi:10.1371/journal.pone.0236197)
Supplement: Attachment — Submitted filename: Reviewers comments_PONE-D-20-15912.docx [file pone.0236197.s010.docx]

**Minor comments:**

In the present study (PONE-D-20-15912) the authors explored to identify novel gastric cancer (GC)-susceptible genes and performed whole-exome sequencing (WES) in 19 GC-affected members and 36 unaffected FDRs of 14 families in which 2 or more GC cases had occurred within the third generation. Linkage and association analyses identified MUC4 missense variants as a predisposition to the familial aggregation of GC. Although, this is a large and well-designed case-control study and authors have performed extensive tools to discover common variants in MUC4 region which are significantly linked with GC, I have following minor comments regarding the study:

1. Sample size calculation for patients with GC and control is not mentioned. For calculation of sample size, which software was used and specify the test used. How the power of the study was calculated? Also, please mention how the effect size of SNPs was analyzed?
2. Inclusion and exclusion criteria for the participants should be mentioned clearly. For example, how the healthy subjects were recruited? Did they fill out questionnaires on gastrointestinal symptoms? For a diagnosis of GC, was it based on one or a combination of clinical, radiological, and endoscopic criteria?
3. Is the genotype frequency in MUC4 gene differ in any particular age group?
4. *H. pylori* status in co-relation with MUC4 variant might have some roles in pathogenesis of GC. The results section lacks association between GC with or without MUC4 variant and *H. pylori* status. Also, some discussion on this point needs to be included.
5. In the introduction section, it should be mentioned on the role of MUC on host physiology, and how mutant variant might affect in the pathogenesis. The roles of MUC4 can only be speculative at this moment without any functional studies.
6. Please mention the clinical history of patients with anti-microbial therapy, H2-receptor blockers etc. were excluded from this study? This will make the study for better presentation.
7. How the variant MUC4 is associated with GC disease severity?
8. Did the authors observe any association of MUC1, and MUC4 variants in a same GC patient? As MUC1variant and gastric carcinogenesis is well documented.
9. What are the study limitations? Addition of a paragraph on this would be better.
10. Though the host's genetic factors play an important role in gastric carcinogenesis, the role of diet cannot be denied. History of dietary habit might add some novel finding in relation with MUC4 variant.
